# Upper limb erythema nodosum: the first presentation of Crohn's disease

**DOI:** 10.1002/ccr3.87

**Published:** 2014-06-30

**Authors:** R E Faulkes

**Affiliations:** Good Hope HospitalRectory Road, Sutton Coldfield, West Midlands, B75 7RR

**Keywords:** Crohn's disease, erythema nodosum, inflammatory bowel disease, upper limb

## Abstract

**Key Clinical Message:**

Inflammatory bowel disease can present with extraintestinal features as the patient's only complaint. The erythema nodosum (EN) initially affected the upper limbs only, reminding us that signs do not always present in a classical fashion. The presence of EN should prompt the clinician to look for any underlying cause.

A 23-year-old female presented to Accident and Emergency (A and E) with a 3-day history of a painful left elbow. It had been red and swollen following mild trauma. She also reported 11 days of diarrhea, which had been diagnosed as viral gastroenteritis at her GP practice following negative stool cultures. She had a past medical history of polycystic ovarian syndrome. She did not take any regular medication and had no significant family history.

On examination she was apyrexial. Her left elbow was erythematous, swollen, and tender with full range of movement. A tender erythematous lesion was also noted at the base of her left thumb. Cardio, respiratory, and abdominal examinations were unremarkable.

Blood tests showed raised WCC and inflammatory markers. X-ray of elbow revealed no bony abnormality or joint effusion. The patient was diagnosed with cellulitis and discharged 4 days later with oral flucloxacillin.

Three days later, the same patient presented again to A and E with diffuse joint pain. Her diarrhea was persisting, now for 3 weeks in total. She was opening her bowels six times per day. On examination, there were raised, tender, erythematous patches over her wrists, elbows, and the anterior aspect of her lower limbs bilaterally. The patient had persistently raised inflammatory markers, with CRP 231 mg/L and platelets 670 × 10^6^/L.

Flexible sigmoidoscopy showed a granular, erythematous sigmoid colon with multiple ulcers consistent with active Crohn's disease, which was confirmed on histology. On further questioning, the patient reported unintentional weight loss of one stone over 3 months and nocturnal diarrhea. She was started on hydrocortisone and mesalazine and responded well to a course of steroids.

## Discussion

Erythema nodosum (EN) is a panniculitis, or inflammation of subcutaneous fat. It is one of the extraintestinal manifestations of inflammatory bowel disease (IBD). There are a number of associated skin lesions in IBD, including pyoderma gangrenosum, aphthous stomatitis, and perianal fissures. However EN is the most common, reported to occur in 2–15% of Crohn's cases 1,2. The presence of EN relates to disease activity, and has a higher incidence in Crohn's than ulcerative colitis (UC) 3. It is also more prevalent in younger patients 4 and in females in the acute stages of disease 5.

It presents as tense, erythematous nodules that become purplish, fluctuant lesions. The lesions progress to a bruise-like appearance before resolving after two to four weeks. They typically occur over the shins, thighs, and forearms 6. EN may be accompanied by systemic symptoms of arthralgia and fever, particularly in adults 7.

Erythema nodosum as the presenting feature of Crohn's disease has been reported elsewhere in the literature, sometimes preceding the diagnosis of IBD for a number of years 8. In one case, a child presented solely with the skin lesions and no bowel symptoms, but had endoscopic features of active disease in the ascending colon and terminal ileum 9.

It has been proposed that the occurrence of EN in Crohn's disease is due to a T-cell mediated response to common antigens between gut bacteria and the skin 2. Cases of EN in IBD have been associated with positive ANCA and HLA B27 10 and variants of the TRAF3IP2 allele 11, suggesting that genetic factors also play a role in determining which patients with IBD develop cutaneous manifestations.

While idiopathic in up to 50% of cases, EN can be due to potentially serious underlying disease, including granulomatous diseases, malignancy (lymphoma and leukemia), and infection. EN may also be drug related (oral contraceptive pill, sulfonamides, phenytoin) and can develop during pregnancy 12. With such a broad range of potential causes, a useful and practical approach to categorizing the etiology is to consider each differential based on the patient's symptoms.

As in the case described above, diarrhea and EN should suggest either Crohn's disease or UC. Less commonly (<1% of cases) it occurs in acute infections including *Campylobacter* and *Salmonella spp*, and may, therefore, present with diarrhea of an infectious, rather than autoimmune, etiology 5.

For the patient with EN and respiratory symptoms, it may be due to tuberculosis or sarcoidosis. In the latter EN is often associated with bilateral hilar lymphadenopathy on chest X-ray. This is known as Löfgren's syndrome and has a good prognosis 13.

EN may also occur in Beçhet's disease, defined as a triad of aphthous ulceration, genital ulcers, and uveitis. Leprosy is another potential, if rare, cause of EN.

Children can develop EN, most commonly following an acute Streptococcal throat infection 7. It has also been reported as the presenting factor of IBD in pediatric patients as well as adults 14.

In all cases, inflammatory markers are usually raised, including in idiopathic EN. Many differentials may be excluded through thorough history taking, which should also guide initial investigations such as chest X-ray, sputum or stool cultures.

Management of EN is primarily to identify and treat the underlying cause. In IBD the clinical course of EN generally correlates to the activity of bowel disease. Successful treatment of Crohn's or UC with steroids, 5-aminosalicylates or immunomodulatory therapy leads to resolution of the skin lesions 2. The successful use of anti-TNF-*α* agents such as adalimumab has been reported for EN and Crohn's resistant to other treatments 15, although it should be noted that a case of EN developing subsequent to the use of infliximab has also been documented in the literature 16.

Idiopathic cases of EN are self-limiting, but are more likely to recur. One case series reported a 62% annual relapse rate of idiopathic lesions 12. Patients may benefit from symptomatic treatment with rest, nonsteroidal anti-inflammatories, and topical application of potassium iodide solution. Steroids may again be considered 6.
